# Editorial Note: Intra-industry peer effect in corporate environmental information disclosure: Evidence from China

**DOI:** 10.1371/journal.pone.0345268

**Published:** 2026-03-19

**Authors:** The PLOS One Editors

The *PLOS One* Editors issue this Editorial Note because this article [1] was identified as one of a series of submissions for which we have concerns about aspects of the peer review process. Readers are advised to interpret the article [1] with caution.

The Editors have no evidence of any author involvement in the peer review concerns.
